# Evolutionary and Mutational Characterization of the First H5N8 Subtype Influenza A Virus in Humans

**DOI:** 10.3390/pathogens11060666

**Published:** 2022-06-08

**Authors:** Lin Ding, Jie Li, Xue Li, Bingqian Qu

**Affiliations:** 1 Department of Cardiology, Shanghai General Hospital, Shanghai Jiaotong University School of Medicine, Shanghai 200240, China; linding598@gmail.com; 2 Department of Basic Medical Sciences & Forensic Medicine, Hangzhou Medical College, Hangzhou 310059, China; 881012021033@hmc.edu.cn; 3 Department of Internal Medicine III, University Hospital Heidelberg, 69120 Heidelberg, Germany; xue.li@med.uni-heidelberg.de; 4 Department of Veterinary Medicine, Paul Ehrlich Institute, 63225 Langen, Germany; 5 European Virus Bioinformatics Center (EVBC), 07743 Jena, Germany

**Keywords:** influenza A virus, high pathogenic avian influenza, H5N8, phylogenetic analysis, tMRCA, mutation, evolution, human transmission

## Abstract

Highly pathogenic influenza A virus H5 subtype remains a risk for transmission in humans. The H5N8 subtype has caused multiple outbreaks in poultry in Europe over the past few winters. During one recent outbreak in poultry in Astrakhan, workers on the farm were also infected. So far, little is known about how this virus evolves and adapts to infect humans. Here, we performed a time-resolved phylogenetic analysis of 129 HA sequences representing all 1891 available H5N8 viruses collected from 2010 to 2020. We also conducted a whole-genome scan on the human virus at the protein level. We found that H5N8 viruses have spilled over in 34 European countries during the flu season of 2020–2021. These viruses underwent two significant evolutionary steps during 2015–2016 and after 2018. Furthermore, we characterized a number of critical mutations in all viral proteins except PB1-F2, which contribute to increased virulence and avian-to-human adaptation. Our findings suggested that the accumulated mutations under evolution led to quantitative and qualitative changes, likely allowing the virus to spread to humans. Given that the H5N8 virus is co-circulating with other H5 viruses in Europe, the risk of a pandemic should not be underestimated. Continental surveillance and pandemic preparedness are to be established.

## 1. Introduction

Influenza A viruses infect a broad range of hosts, and have the potential to cause pandemics. They are classified into subtypes based on the combination of hemagglutinin (HA: H1 to H18) and neuraminidase (NA: N1 to N11) surface glycoproteins. A virion enveloped with HA and NA contains eight segmented RNA genomes. Highly pathogenic avian influenza (HPAI) A viruses, mostly H5 or H7 subtypes, have crossed species barriers, and have caused sporadic infections in humans with high fatality rates [1]. A precedent H5N1 virus (A/Goose/Guangdong/1/1996 (Gs/Gd/96) appeared in farmed geese in southern China. Its descendants underwent subsequent reassortment with co-circulating H9N2 avian viruses and, for the first time, transmitted from poultry to humans in Hong Kong in 1997 [2,3]. The Gs/Gd/96 lineage has continued to evolve and circulate in multiple host species for more than two decades, and these homologous HA genes have diversified various clades and subclades.

The H5N8 clade 2.3.4.4 was initially identified in poultry during 2009–2010 in mainland China [4]. Later, in an outbreak in Korea in 2014, the lineage was subsequently classified into two clades: 2.3.4.4a (A/broiler duck/Korea/Buan2/2014 and A/Baikai teal/Korea/Donglim3/2014) and 2.3.4.4b (A/breeder duck/Korea/Gochang1/2014) [5]. They then spread further to Europe, Africa, and North America, probably via migratory wild birds [6,7,8]. By November 2014, nine outbreaks of these H5N8 viruses occurred in poultry among four countries in Europe. The viral strains were similar to cluster A (Bu-an2/Donglim3-like) in Korea [9]. Independently of this, the 2.3.4.4b H5N8 virus underwent reassortment with other circulating Eurasian avian influenza viruses. The reassortant viruses showed better adaptation, spilled over in wild birds and poultry, and caused the largest documented H5N8 epidemic in Europe during the flu season of 2016–2017 [10]. Although the reassortant became one of the dominant viruses present in wild waterfowl reservoirs, the infection in humans was not reported.

When HPAI H5N8 outbreaks appeared in poultry in the winter of 2020, workers on a poultry farm in the Astrakhan Oblast region of the Russian Federation were confirmed to be H5 influenza positive. One virus isolated from a 28-year-old female worker was identified as subtype H5N8, A/Astrakhan/3212/2020(H5N8). Meanwhile, in December 2020, other H5N8 viruses, A/chicken/Astrakhan/321-01/2020(H5N8) and A/chicken/Astrakhan/2171-1/2020(H5N8), were also isolated from chickens on the same farm, where the infected individuals worked [11]. To date, it is the first report that an H5N8 virus can cross the species barrier and infect humans. Before this case, H5N8 viruses had been reported to infect other mammalian hosts, including domestic and wild pigs, foxes, and gray seals [12,13,14,15]. Although all infected individuals were asymptomatic, the World Health Organization emphasized the demand for further risk assessment towards pandemic preparedness. However, so far, little is known about this emerging viral strain.

We showed a map of H5N8 outbreaks in European countries over the past four flu seasons, and observed significantly increased cases in winter 2020–2021. To decipher how different the human virus compares with other H5N8 viruses, we performed phylogenetic analyses on all 8 segments, and dissected 129 representative HA sequences from 1891 H5N8 virus strains collected between 2010 and 2020 in Asia, Africa, and Europe. Using the most recent common ancestor (tMRCA) computation, we revealed that the Astrakhan H5N8 viruses from humans and chickens shared a probable ancestor after 2018 in the molecular evolutionary clock. Furthermore, mutations on the amino acid level in PB2, PB1, PA, NP, and non-structural (NS1) genes are likely responsible for human adaptation.

## 2. Results

### 2.1. GISAID Database Recorded H5N8 Outbreaks in Europe during 2017–2021

**Figure 1:**
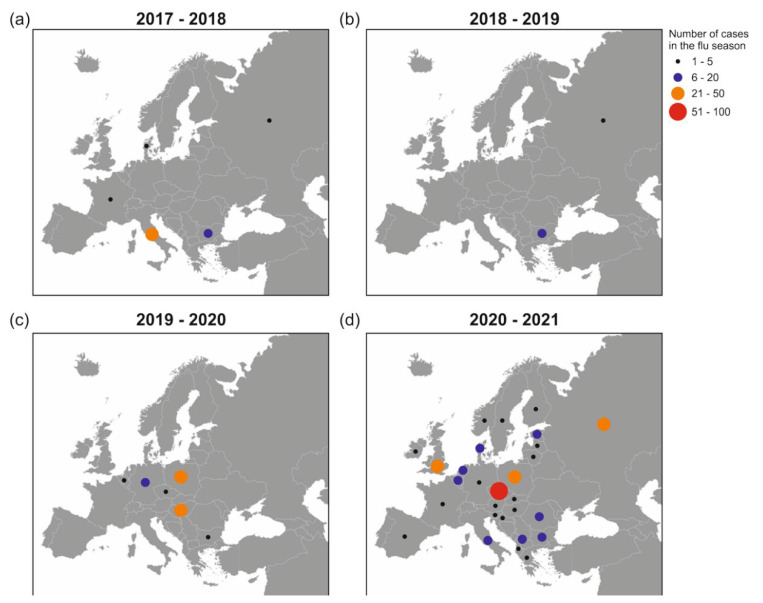
GISAID database recorded H5N8 outbreaks over the past four flu seasons in Europe. The circle labeled on each country indicates the number of cases in the flu seasons from (**a**) 1 October 2017 to 31 May 2018, (**b**) 1 October 2018 to 31 May 2019, (**c**) 1 October 2019 to 31 May 2020, and (**d**) 1 October 2020 to 31 May 2021. The size and color of the circles show the numbers: small black—1 to 5; medium blue—6 to 20; large orange—21–50; large red—51–100. Raw map credit is distributed under the Creative Commons license (attribution 3.0) (http://freedesignfile.com, accessed on 24 January 2022).

How H5N8 viruses evolved and spread before 2016 has been studied [7]. Here, we focused on the events after 2016, and analyzed the H5N8 cases recorded in the GISAID database in 34 countries in Europe during each flu season since 2017. The accurate number of events in each year and country was not available or clearly mentioned in other databases. The virus caused an outbreak in Italy and some sporadic cases in four countries during 2017–2018. The next year, it was merely documented in Romania and the Russian Federation. During the winter of 2019, it spread in mid-eastern Europe, Hungary, and Poland, with individual cases emerging in Germany, the Czech Republic, Romania, and the Netherlands. During 2020–2021, H5N8 viruses not only spilled over in hotspots such as the Czech Republic, Poland, the United Kingdom, and the Russian Federation, but also spread to 28 countries on the continent (Figure 1). Of note, there was no evident spatial association between virus distribution and poultry population density in Europe. The actual number of cases may be higher than that shown in this figure. Still, these numbers over four flu seasons were comparable, and genomic information of each virus causing an outbreak can be tracked in the GISAID database.

### 2.2. Human-Origin and Chicken Viruses Are Closely Related but Not Homologous to an Earlier Virus in Astrakhan

**Figure 2:**
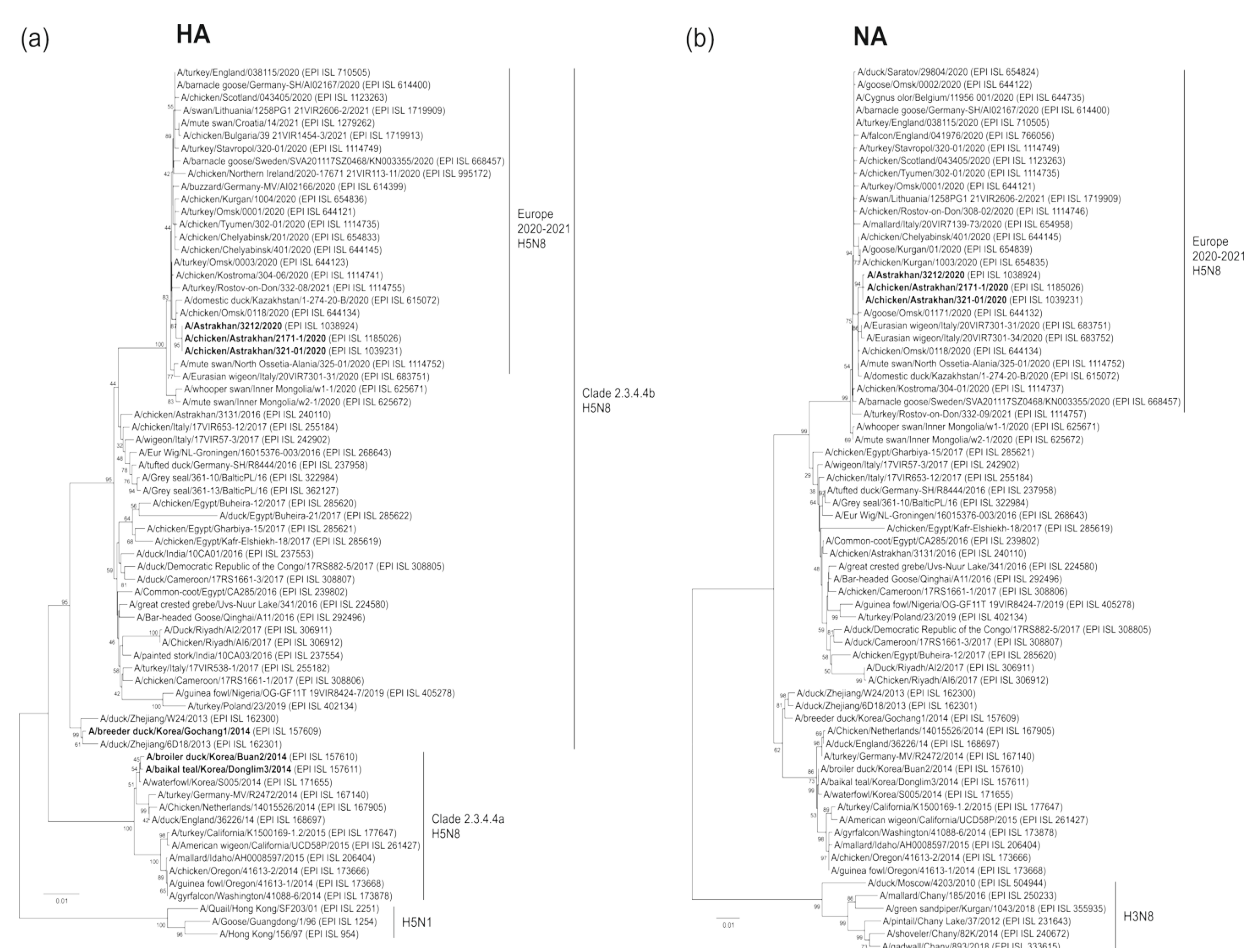
Phylogenetic trees for HA and NA segments of influenza A H5N8 viruses isolated in Europe from 2014 to 2021. (**a**) HA and (**b**) NA sequences of European H5N8 viruses, reference H5N8 viruses (Gochang1, Buan2, and Donglim3, highlighted) and other subtype references (HA of H5N1 viruses in (**a**), NA of H3N8 viruses in (**b**), respectively) were analyzed. Clades of H5N8 viruses in Europe in 2020–2021, 2.3.4.4a and 2.3.4.4b, are marked. The six digits following the EPI ISL are identity numbers in the GISAID EpiFlu database. Scale bars indicate the number of nucleotide substitutions per site. The numbers on the nodes are bootstrap values.

**Figure 3:**
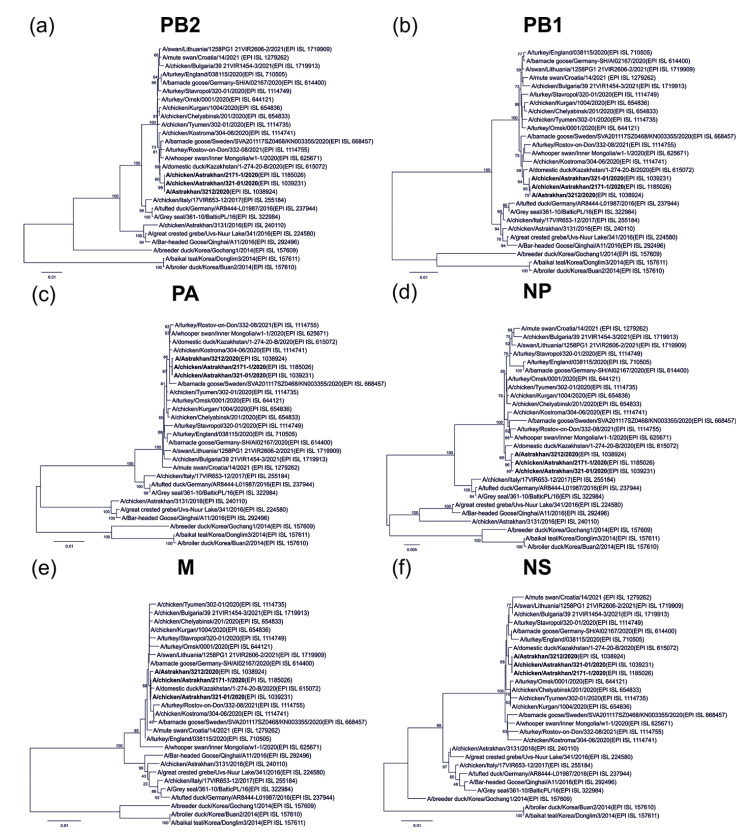
Phylogenetic trees for six internal genes: (**a**) PB2; (**b**) PB1; (**c**) PA; (**d**) NP; (**e**) M, and (**f**) NS. Human and chicken Astrakhan viruses in 2020 are highlighted. The six digits attached to the virus name are identity numbers in the database. Scale bars indicate the number of nucleotide substitutions per site. Bootstrap values are shown on the nodes of each tree.

Astrakhan is located at the delta where the Volga River joins the Caspian Sea, a natural habitat for wild birds. It is a part of the Black Sea/Mediterranean and east Africa/west Asia migration routes, and provides a breeding ground for viral reassortment [8]. To evaluate viral reassembly, we executed phylogenetic analyses. Our data demonstrated that HA sequences of human and chicken Astrakhan viruses in 2020 were closely related. All three viruses in this cluster belonged to the H5 clade 2.3.4.4b. They were homologous to other H5N8 viruses isolated in Omsk, Rostov-on-Don, Russia, and in neighboring Kazakhstan (Figure 2a). Overall, three Astrakhan viruses shared similar NA segments closely related to those from Omsk and Italy (Figure 2b). A total of six internal genes of the human H5N8 virus were most closely associated with two chicken viruses, indicating a chicken-to-human transmission. These segments were also homologous to those of other Eurasian viruses in 2020. Remarkably, none of these Astrakhan viruses in 2020 came close to an earlier Astrakhan virus in 2016 (A/chicken/Astrakhan/3131/2016(H5N8)) (Figure 3a–f).

### 2.3. H5N8 Virus Took Two Evolutionary Steps during 2015–2016 and after 2018

**Figure 4:**
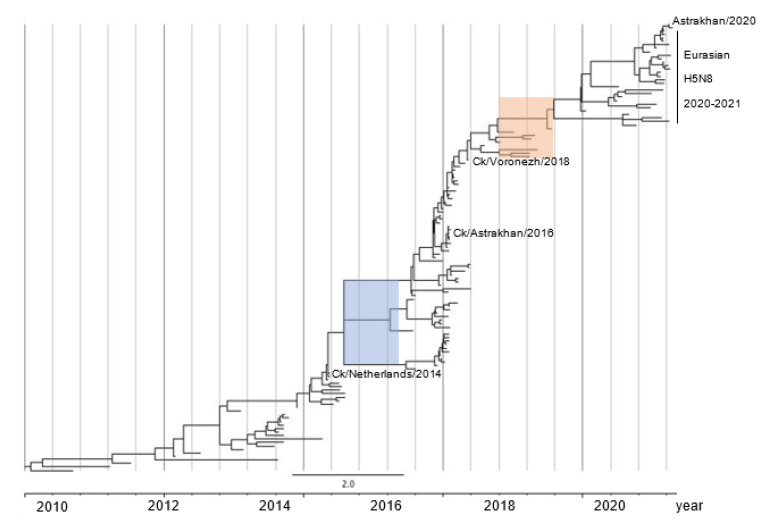
Estimation of a time-resolved phylogenetic tree containing 129 HA genes using a Bayesian Markov Chain Monte Carlo approach. The layout was visualized using FigTree software. Each branch stands for the HA gene of one H5N8 virus with the exact collection time shown in the GISAID database. Astrakhan viruses in 2016 and 2020 are marked. Blue rectangle: first major evolutionary step. Orange rectangle: second major step. The scale bar equates to two years and the interval between vertical lines is 0.5 year. Year information is shown beneath the bottom timeline. Highlighted viruses: Ck/Netherlands/2014: A/chicken/Netherlands/14015526/2014(H5N8); Ck/Voronezh/2018: A/chicken/Voronezh/1488/2018(H5N8); Ck/Astrakhan/2016: A/chicken/Astrakhan/3131/2016(H5N8). Astrakhan/2020: A/Astrakhan/3212/2020(H5N8, human).

To estimate the time of occurrence, we selected 129 HA sequences of the human H5N8 virus representing all 1891 available H5N8 sequences from Asia, Africa, and Europe over the last decade. Next, we performed a tMRCA analysis using BEAST software. The time-resolved phylogenetic tree for the HA segments revealed two important evolutionary forward steps. First, H5N8 viruses, including A/chicken/Astrakhan/3131/2016(H5N8), migrated at the node of the ancestral virus lineage A/chicken/Netherlands/14015526/2014(H5N8), and evolved divergently into three clusters. Consistent with a previous study, this evolutionary event caused the epidemic in 2016, when H5 viruses deluged wild birds and spread to Europe and Africa via migratory flyways [10]. Second, one cluster took a further evolutionary step from a common ancestor in 2018, likely A/chicken/Voronezh/1488/2018(H5N8) (Figure 4 and Table S1). We could not perform a refined analysis to deduce what happened during 2018–2019 due to a limited number of complete HA sequences present in the GISAID database at this interval. One possible reason explaining the lower number of viruses recorded during 2018–2019 is that they did not have a major antigenic difference compared to earlier viruses. It remains unclear how the second step proceeded after 2018, but this resulted in the outburst in the winter of 2020, as shown in Figure 1d.

### 2.4. Molecular Features of HA and NA Proteins of the Human H5N8 Virus

**Figure 5:**
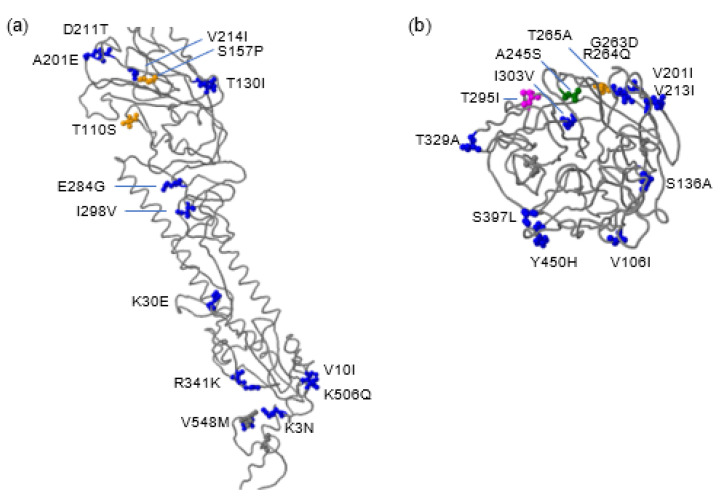
Mutational analysis of the human H5N8 virus. (**a**) HA proteins of A/Astrakhan/3212/2020 and A/Baikal teal/Korea/Donglim/3/2014 were compared, and the result was shown in a structural model of A/Viet Nam/1203/2004(H5N1) (PDB ID 3FKU) [18]. T110S and S157P mutations are marked in yellow, and other mutations are shown in blue. (**b**) Two NA proteins were compared. The template of A/duck/Ukraine/1/63(H3N8) (PDB ID 4GB1) was used to model the NA proteins [19]. Green: A245S; Yellow: T265A; Magenta: T295I; Blue: other mutations. All residue numbers are the actual number in the Donglim3 reference virus.

Next, we investigated the HA and NA protein sequences of the human H5N8 virus using the FluSurver tool, which highlights candidate mutations regarding any predicted phenotypes. We compared A/Astrakhan/3212/2020 with A/Baikal teal/Korea/Donglim/3/2014 as a reference. Although the human virus developed 17 mutations in the HA protein during its evolution, no changes were found at the receptor-binding site. This is consistent with the unlikelihood of human-to-human transmission, as none of the infected person‘s relatives were influenza-positive. However, the HA protein of the human virus has acquired a T110S substitution associated with host specificity shift and an S157P substitution with enhanced antigenic drift (Figure 5a). The virus has also 137A, 158N, 160A, and 186N residues in its HA protein, which have been reported to increase the affinity to α–2,6 sialic acids, a prerequisite for efficient human transmission. In addition, there are multiple mutations in the NA protein. A245S and T265A may lead to stronger oseltamivir and zanamivir resistance [16,17] (Figure 5b). Both Astrakhan chicken viruses in 2020 carried the same mutations, since they had identical HA and NA proteins to the human virus.

### 2.5. Probable Human Adaptive Mutations Are Identified in Astrakhan H5N8 Viruses in 2020

Transmission of the H5N8 virus from chickens to humans provided an opportunity to investigate critical adaptive mutations by comparing viruses isolated from both species. Therefore, we conducted a whole-genome analysis of viral proteins of human and chicken Astrakhan viruses in 2020, as well as six chicken viruses isolated during 2014–2020, in terms of their phylogenetic difference. Molecular changes in residues are listed in Table 1. All viruses possessed a furin cleavage site (REKRRKR↓GLF) in the HA protein, showing a high pathogenicity phenotype in chickens. Human-to-human transmission appears to be limited, since the virus had QRG but not LRS residues at the receptor-binding site. Compared to other viruses, the human virus has two specific mutations: PA-A598T and NA-S29N (S28N in the N8 numbering). Furthermore, three Astrakhan viruses in 2020 had mutations in multiple proteins: PB2-N556S, PB2-E677G, PB1-E614D, PA-N115D, NP-G16S, NP-A129S, NA-V51I, and NA-M296I. Notably, we found that, compared to viruses circulating during 2014–2018, all four viruses in 2020 had accumulated mutations in every protein except PB1-F2, e.g., T139A mutation in the M1 protein (Table 1). These substitutions in viral proteins may allow the virus to be more adaptative in mammalian hosts. More validations by reverse genetics approach are required to investigate which mutations can enhance virulence and promote its replication in humans.

## 3. Discussion

Using tMRCA analysis, we showed that European H5N8 viruses had undergone two major evolutionary steps. The first occurred before the European H5 viruses spilled over in 2016. A total of three clusters existed simultaneously during 2015–2016, as shown in the time-resolved phylogenetic tree, but merely one evolved forward (Figure 4). This branch contains the viruses including A/chicken/Astrakhan/3131/2016(H5N8). Second, some of them evolved further after 2018, and developed a number of new mutations, such as T139A in the M1 protein, as shown in Table 1 and Table S2, which resulted in more efficient replication in its natural host, likely wild birds [20].

Over the years, the accumulated mutations in its genome resulted in quantitative and qualitative changes, and eventually spread to humans. Using tMRCA analysis, we found that the novel human H5N8 virus is highly homologous to viruses circulating in birds in Astrakhan and the neighboring Omsk and Rostov-on-Don regions. HPAI H5N8 viruses led to 10.9% mortality in farm chickens. However, they caused asymptomatic infection in humans, suggesting that the virus has not yet adapted to humans [11]. Human-to-human transmission is impractical, since all of their relatives had negative results for the flu tests. On a molecular basis, the human virus lacks Q226L and G228S mutations in the receptor-binding site (Table 1). This suggests that the new virus retains weak binding activity to α–2,6 sialic acid receptor, which is highly expressed in the human airway epithelia [21]. However, T110S and S157P mutations in the HA protein increased receptor binding activity to α–2,3 sialic acids, and enhanced evasion of neutralizing antisera against clade 1 and 2.2 viruses [22,23]. This finding explains why these new viruses can efficiently infect chickens and even humans with pre-existing immunity induced by vaccination. All Astrakhan viruses in 2020 had 137A/158N/160A/186N residues in their HA proteins, which have been reported to increase the affinity to α–2,6 sialic acids. A recent study showed that new H5N6 reassortants bearing the HA gene of H5N8 virus clade 2.3.4.4b caused multiple human infections [24]. This virus generated in nature instead of a reverse genetically engineered virus in the laboratory demonstrated that both H5N8 and H5N6 viruses carrying the HA gene can mediate human infections. The HA protein of the Astrakhan viruses binds preferentially α–2,3 sialic acids, but likely has an increased affinity to α–2,6 sialic acids.

The NS1 protein is considered one of the major virulence determinants. Compared to earlier viruses, the viruses in 2020 carried 12 mutations, which contributed to enhanced type I interferon antagonistic properties, leading to higher virulence in ferrets (Table 1) [25]. This finding is consistent with a recent publication showing that the C-terminal NS1 in H5N8 2.3.4.4 clade regulates viral fitness in human cells and virulence in mice [26]. We did not observe E627K and D701N mutations in the PB2 protein responsible for enhanced virulence [27]. However, critical mutations present in all three viral polymerase subunits (PB2-N556S, PB2-E677G, PB1-E614D, PA-N115D, and PA-A598T) provide a message that H5N8 viruses in 2020 may have a different activity in viral replication. The substitutions that emerged in other viral proteins e.g., PA-A598T and NA-S29N, may allow the virus a better human-specific adaptation after infection.

Little is known about drug resistance in the human virus, since all the people who tested H5N8 positive were self-recovered without medication. The A245S mutation was identified, while the R292K mutation in the NA protein was not found (Figure 5 and Table S2). This variant was resistant to oseltamivir (Tamiflu), but remained susceptible to zanamivir (Relenza) and peramivir [28]. The T265A mutation is present only in 0.44% of all known influenza viruses. This mutation may also be related to resistance to neuraminidase inhibitors [16,29]. Mutations N46K and T295I remove potential N-glycosylation sites at positions 44 and 293, respectively, which may affect the antigenicity of the human virus.

At this stage, a major limitation is that serological data are not sufficient to determine the antigenicity of the human H5N8 virus. The important link between genetic and antigenic evolution was not clear either. Overall, two scientific questions were not answered in the first publication [11]. First, whether the human H5N8 virus can be neutralized by antisera against other H5 viruses, such as H5N1, H5N2, and H5N6. Second, whether H5N8 antisera in infected individuals have a neutralizing activity to above H5 viruses. Therefore, an in-depth global collaboration on H5N8 viruses is urgently required to better understand how efficient this new virus infects and replicates in human epithelial cells.

In conclusion, a new H5N8 virus has infected humans, despite no evidence of human-to-human transmission. H5N8 viruses have impacted Europe twice in the past five years. So far, they circulate and co-exist with other H5 viruses. We should not underestimate the risk that some viruses can cause epidemics in humans, such as what we have learned from the course of others, such as the H7N9 epidemic in 2013. Surveillance and pandemic preparedness of the novel H5N8 viruses should be strengthened in Europe [30].

## 4. Materials and Methods

### 4.1. Viral Strains

If not stated otherwise, all sequences used in this study are online available in the GISAID database [31]. Pyankova et al. isolated A/Astrakhan/3212/2020 (GISAID accession number EPI_ISL_1038924) from a 28-year-old female worker, and propagated it in the Madin–Darby canine kidney (MDCK) cells, whereas A/chicken/Astrakhan/321-01/2020 (EPI_ISL_1039231) and A/chicken/Astrakhan/2171-1/2020 (EPI_ISL_1185026) were obtained by passaging avian samples in embryonated chicken eggs. Whole genomes of the above viruses were determined using next-generation sequencing [11]. A/chicken/Rostov-on-Don/308-02/2020 (EPI_ISL_1114746), A/chicken/Voronezh/1488/2018 (EPI_ISL_336929), A/chicken/Rostov-on-Don/44/2017 (EPI_ISL_275287), A/chicken/Voronezh/18/2017 (EPI_ISL_247718), A/chicken/Astrakhan/3131/2016 (EPI_ISL_240110), and A/chicken/Netherlands/14015526/2014 (EPI_ISL_167905) were also studied. EPI numbers and details (name, host, collection data, and originating laboratory) of 129 H5N8 viruses used in the tMRCA analysis are summarized in Table S1.

### 4.2. Phylogenetic Analysis

HA segments from the emerging H5N8 viruses during 2014–2021 were downloaded from the GISAID database. As shown in Figure 2a, we also analyzed three H5N1 viruses, including ancestral Gs/Gd/96, the first human virus and a quail virus. Those sequences in FASTA format were aligned using the ClustalW tool embedded into the MEGA 6.0 software [32]. The result was exported in MEGA format for tree construction. A phylogenetic tree was further built using a neighbor-joining algorithm with one thousand bootstrap replicates. For NA analysis, the H5N8 viruses isolated from 2014 to 2021 and six H3N8 viruses found in the Russian Federation were aligned and reconstituted in a phylogenetic tree (Figure 2b). For six internal genes, each segment of three Astrakhan H5N8 viruses mentioned above was compared with those of the Eurasian strains during 2014–2021 (Figure 3).

### 4.3. Time to Most Recent Common Ancestor (tMRCA) Analysis

To investigate the latest common ancestor of the Astrakhan H5N8 viruses in 2020 on an evolutionary clock, we pre-selected all available 1891 complete HA sequences in the GISAID database (161 from Africa, 847 from Asia, and 884 from Europe). A total of 32 available HA sequences from the American continent were excluded in this analysis, since these H5N8 viruses in North America form a cluster belonging to clade 2.3.4.4a, distinct from the European clade, as shown in Figure 2a. Redundant sequences, either with identical sequence information or in the same outbreak, were excluded. Subsequently, 129 HA sequences representing all 1891 original viruses were downloaded from the GISAID database in FASTA format. Each sequence in this special format contains the collection date. The FASTA file was imported into the MEGA 6.0 software, aligned with the ClustalW and exported as NEXUS format. This layout was imported into the Bayesian Evolutionary Analysis Utility (BEAUti) module in the Bayesian Evolutionary Analysis Sampling Trees (BEAST) 2.5.2 module, which provides the site model, clock model, and the Markov Chain Monte Carlo (MCMC) methods. All parameters in these methods were optimized [33]. The result was saved as XML files, and further computed by the BEAST tool. Log file and tree file layouts were examined by Tracer and TreeAnnotator tools to ensure all parameters were sufficiently optimized. Finally, the tree file was visualized by the FigTree 1.4.4 software (http://tree.bio.ed.ac.uk/software/figtree, accessed on 17 May 2021).

### 4.4. Mutational Analysis with the FluSurver to an Ancestral H5N8 Strain

We imported the protein sequence of the HA coding region of A/Astrakhan/3212/2020 (GISAID accession number EPI1846961) into the FluSurver for real-time surveillance of influenza mutations (https://flusurver.bii.a-star.edu.sg, accessed on 24 January 2022, and compared it with the HA protein of A/Baikai teal/Korea/Donglim3/2014(H5N8). A series of mutations were listed on the structural backbone of the Donglim3 reference. The NA protein sequence of A/Astrakhan/3212/2020 (accession number EPI1846963) was also compared with that of the Donglim3, and analyzed in the same server.

### 4.5. Molecular Comparison between Human and Avian H5N8 Viruses in Astrakhan

To decipher the molecular difference between the emerging viruses in 2020 and those isolated earlier in the same region, we aligned each viral protein (PB2, PB1, PB1-F2, PA, HA, NP, NA, M1, M2, NS1, and NS2) of A/Astrakhan/3212/2020, A/chicken/Astrakhan/321-01/2020, and A/chicken/Astrakhan/2171-1/2020 with six chicken viruses isolated during 2014–2020. Critical residues, including those involved in virulence, receptor binding and drug resistance as previously described, were compared and listed in Table 1 and Table S2 [34].

## Figures and Tables

**Table 1:** Molecular characteristics of human and chicken H5N8 viruses during 2014–2020.

	Virus (Year)	Ak/3212/ 2020	Ck/Ak/321-01/ 2020	Ck/Ak/2171-1/ 2020	Ck/RoD/308-02/ 2020	Ck/V/1488/ 2018	Ck/RoD/44/ 2017	Ck/V/18/ 2017	Ck/Ak/3131/ 2016	Ck/NL/14015526/ 2014
	**\Host** **Residue**	human	chicken	chicken	chicken	chicken	chicken	chicken	chicken	chicken
**PB2**	354	V	V	V	V	I	I	I	I	I
	356	I	I	I	I	V	V	V	V	V
	522	H	H	H	H	Q	Q	Q	Q	L
	556	S	S	S	N	N	N	N	N	N
	677	G	G	G	E	E	E	E	E	E
**PB1**	142	S	S	S	S	A	A	A	A	A
	181	V	V	V	V	I	I	I	I	I
	384	P	P	P	P	S	S	S	S	S
	614	D	D	D	E	E	E	E	E	E
**PB1-F2**	del.	1-38 aa	1-38 aa	1-38 aa	1-38 aa	1-38 aa	1-38 aa	1-38 aa	1-38 aa	No
**PA**	115	D	D	D	N	N	N	N	N	N
	224	A	A	A	A	S	S	S	S	S
	343	S	S	S	S	A	A	A	A	V
	545	V	V	V	V	I	I	I	I	I
	598	T	A	A	A	A	A	A	A	A
**HA**	236	D	D	D	D	N	N	N	N	N
	522	A	A	A	A	V	V	V	V	V
	CS	REKRRKR↓GLF	REKRRKR↓GLF	REKRRKR↓GLF	REKRRKR↓GLF	REKRRKR↓GLF	REKRRKR↓GLF	REKRRKR↓GLF	REKRRKR↓GLF	RERRRKR↓GLF
	RBS	QRG	QRG	QRG	QRG	QRG	QRG	QRG	QRG	QRG
**NP**	16	S	S	S	G	G	G	G	G	G
	129	S	S	S	A	A	A	A	T	A
	201	V	V	V	V	I	I	I	I	I
	219	F	F	F	F	Y	Y	Y	Y	Y
	371	I	I	I	I	M	M	M	M	M
**NA**	stalk del.	No	No	No	No	No	No	No	No	No
	28	L	L	L	L	V	V	V	V	V
	29	N	S	S	S	S	S	S	S	S
	51	I	I	I	V	V	V	V	V	V
	107	I	I	I	I	V	V	V	V	V
	202	I	I	I	I	V	V	V	V	V
	214	I	I	I	I	V	V	V	V	V
	246	S	S	S	S	A	A	A	A	A
	266	A	A	A	A	T	T	T	T	T
	296	I	I	I	M	T	T	T	T	T
	360	M	M	M	M	V	V	V	V	V
	470	G	G	G	G	K	K	K	K	K
**M1**	139	A	A	A	A	T	T	T	T	T
	248	L	L	L	L	M	M	M	M	M
**M2**	12	K	K	K	K	R	R	R	R	R
	18	N	N	N	N	K	K	K	K	R
**NS1**	del.	218-237 aa	218-237 aa	218-237 aa	218-237 aa	218-237 aa	218-237 aa	218-237 aa	218-237 aa	No
	3	P	P	P	P	S	S	S	S	S
	48	T	T	T	T	N	N	N	N	S
	53	S	S	S	S	G	G	G	G	D
	56	A	A	A	A	T	T	T	T	T
	60	E	E	E	E	A	A	A	A	A
	66	K	K	K	K	E	E	E	E	E
	70	K	K	K	K	E	E	E	E	G
	84	G	G	G	G	S	S	S	S	V
	109	R	R	R	R	Q	Q	Q	Q	Q
	114	P	P	P	P	S	S	S	S	S
	189	N	N	N	N	D	D	D	D	D
	207	D	D	D	D	N	N	N	N	N
**NS2**	3	P	P	P	P	S	S	S	S	S
	31	I	I	I	I	M	M	M	M	M

Abbreviations: aa: amino acids; Ak: Astrakhan; Ck: chicken; CS: cleavage site; del.: deletion; NL: Netherlands; RBS: receptor-binding site (residues 222~224 in the H5 or 226~228 in the H3 numbering); RoD: Rostov-on-Don; V: Voronezh. The residues are marked if they are mutated (1) only in the human virus or (2) in three Astrakhan viruses in 2020, or (3) in all four viruses in 2020. (↓: cleavage site).

## Data Availability

The data are available from the corresponding author upon request.

## Supplementary Materials

The following is available online at https://www.mdpi.com/article/10.3390/pathogens11060666/s1, Table S1: Details of 129 H5N8 viruses in the GISAID database used in the tMRCA analysis. Table S2: Molecular comparison between Astrakhan and other H5N8 viruses during 2014–2020.
