# Supplementary material for: Evolutionary and Mutational Characterization of the First H5N8 Subtype Influenza A Virus in Humans
Source: Pathogens. 2022 Jun 8;11(6):666. doi: 10.3390/pathogens11060666 (PMC9227545; doi:10.3390/pathogens11060666)
Supplement: Supplementary file 1 [file pathogens-11-00666-s001.zip › Table S2.pdf]

Table S2. Molecular comparison between Astrakhan viruses and other H5N8 viruses during 2014-2020.

	**Virus**	**Ak/3212/2020**	**Ck/Ak/321-01/2020**	**Ck/Ak/2171-1/2020**	**Ck/RoD/308-02/2020**	**Ck/V/1488/2018**	**Ck/RoD/44/2017**	**Ck/V/18/2017**	**Ck/Ak/3131/2016**	**Ck/NL/14015526/2014**
**Protein**	**\Host Residue**	human	chicken	chicken	chicken	chicken	chicken	chicken	chicken	chicken
**PB2**	4	I	I	I	I	I	T	I	I	I
	66	M	M	M	M	M	M	M	I	M
	67	I	I	I	I	I	I	I	I	V
	82	N	N	N	N	S	N	N	N	N
	89	V	V	V	V	V	V	V	V	V
	106	T	T	T	T	T	T	T	T	A
	109	V	V	V	A	V	V	V	V	V
	139	V	V	V	V	V	V	V	V	I
	176	I	I	I	I	V	I	I	I	I
	187	R	R	R	R	K	K	K	R	K
	199	A	A	A	A	T	A	A	A	A
	261	I	I	I	I	I	V	I	I	I
	292	V	V	V	V	V	V	V	V	T
	338	V	V	V	V	V	V	V	V	I
	339	K	K	K	K	K	K	K	K	R
	340	R	R	R	R	R	R	R	K	K
	344	I	I	I	I	I	V	V	V	V
	354	V	V	V	V	I	I	I	I	I
	356	I	I	I	I	V	V	V	V	V
	453	P	P	P	P	L	P	P	P	P
	508	R	R	R	K	K	R	R	R	Q
	522	H	H	H	H	Q	Q	Q	Q	L
	556	S	S	S	N	N	N	N	N	N
	559	T	T	T	T	T	T	T	T	I
	575	M	M	M	M	M	M	M	M	V
	611	D	D	D	D	D	D	D	N	D
	613	V	V	V	V	V	V	V	V	I

	617	K	K	K	K	K	K	K	R	K
	627	E	E	E	E	E	E	E	E	E
	628	Q	Q	Q	Q	Q	Q	Q	Q	H
	630	R	R	R	R	R	R	R	K	R
	666	T	T	T	T	T	T	T	T	A
	667	V	V	V	V	V	V	V	I	V
	677	G	G	G	E	E	E	E	E	E
	678	Y	Y	Y	Y	Y	Y	Y	D	D
	684	A	A	A	A	A	A	A	S	T
	699	K	K	K	K	K	K	K	K	R
	701	D	D	D	D	D	D	D	D	D
	702	K	K	K	K	K	K	K	K	R
	757	A	A	A	A	A	A	A	A	V
**PB1**	57	T	T	T	T	T	T	T	T	K
	99	H	H	H	H	H	H	H	H	H
	110	A	A	A	A	A	A	A	A	T
	113	I	I	I	I	I	V	V	V	I
	142	S	S	S	S	A	A	A	A	A
	168	R	R	R	R	R	R	R	R	K
	181	V	V	V	V	I	I	I	I	I
	215	R	R	R	R	R	R	R	R	K
	216	N	N	N	S	S	S	S	S	N
	219	I	I	I	I	I	I	I	I	V
	302	I	I	I	V	I	I	I	I	I
	317	M	M	M	M	M	M	M	M	I
	361	S	S	S	S	S	N	S	S	S
	368	I	I	I	I	I	I	I	I	I
	374	T	T	T	T	T	T	T	T	A
	375	N	N	N	N	N	N	N	N	T
	384	P	P	P	P	S	S	S	S	S
	398	D	D	D	D	D	D	D	D	E
	569	Q	Q	Q	Q	Q	Q	H	Q	Q
	571	R	R	R	R	K	R	R	R	R

	591	I	I	I	I	I	V	V	V	V
	614	D	D	D	E	E	E	E	E	E
	633	S	S	S	S	S	N	N	S	S
	645	V	V	V	V	I	V	V	V	V
	655	M	M	M	M	M	M	I	M	M
	667	I	I	I	I	I	I	I	I	V
	694	S	S	S	S	S	S	S	S	N
	740	F	F	F	F	L	F	F	F	F
	744	M	M	M	M	M	M	L	M	M
	753	L	L	L	L	L	L	L	L	I
**PB1-F2**	del.	1-38 aa	1-38 aa	1-38 aa	1-38 aa	1-38 aa	1-38 aa	1-38 aa	1-38 aa	No
	43	Q	Q	Q	Q	Q	Q	Q	Q	L
	45	T	T	T	T	T	T	T	T	I
	46	M	M	M	M	M	M	M	T	T
	54	L	L	L	L	L	L	L	L	R
	56	A	A	A	A	A	A	A	A	V
	66	N	N	N	N	N	N	N	N	S
	71	Y	Y	Y	Y	Y	Y	Y	Y	S
	75	L	L	L	L	L	L	L	L	H
	79	R	R	R	R	R	R	R	R	Q
	82	S	S	S	S	S	S	S	S	L
	84	S	S	S	S	S	S	S	S	N
**PA**	20	A	A	A	A	A	A	A	A	T
	27	D	D	D	D	D	D	D	D	N
	36	A	A	A	A	T	A	A	A	A
	59	E	E	E	E	E	E	E	G	K
	96	N	N	N	N	N	N	N	H	N
	100	V	V	V	V	V	V	V	V	V
	101	E	E	E	E	E	E	E	E	D
	113	R	R	R	R	R	R	R	K	K
	115	D	D	D	N	N	N	N	N	N
	127	V	V	V	V	A	V	V	V	V
	129	I	I	I	I	I	I	I	I	T

	162	T	T	T	T	T	T	T	T	I
	184	N	N	N	N	N	S	S	N	N
	216	D	D	D	D	D	Y	D	D	D
	224	A	A	A	A	S	S	S	S	S
	237	E	E	E	E	E	E	E	E	K
	242	I	I	I	I	I	I	I	I	L
	261	L	L	L	L	L	L	L	L	M
	268	L	L	L	L	I	L	L	L	L
	308	T	T	T	T	T	I	I	I	I
	312	K	K	K	R	K	K	K	K	K
	327	E	E	E	E	E	E	E	E	V
	343	S	S	S	S	A	A	A	A	V
	356	K	K	K	K	K	K	K	K	K
	386	D	D	D	D	E	D	D	D	D
	400	S	S	S	S	S	S	S	S	P
	404	A	A	A	A	A	A	A	A	S
	409	S	S	S	S	S	S	S	S	S
	472	A	A	A	A	A	A	A	A	S
	492	K	K	K	K	K	R	K	K	K
	545	V	V	V	V	I	I	I	I	I
	561	M	M	M	M	V	M	M	M	M
	598	T	A	A	A	A	A	A	A	A
	615	R	R	R	R	R	R	R	K	K
	618	T	T	T	T	A	T	T	T	T
	631	G	G	G	G	G	G	G	G	S
	672	L	L	L	L	L	L	L	L	F
	682	D	D	D	D	N	D	D	D	D
	683	L	L	L	L	I	L	L	L	L
	700	V	V	V	V	V	V	V	A	V
**HA**	14	E	E	E	E	E	E	E	E	K
	38	N	N	N	N	N	N	N	N	T
	72	R	R	R	R	R	R	S	S	R
	94	S	S	S	S	S	S	S	S	T

	97	D	D	D	D	N	D	D	D	D
	114	I	I	I	I	I	I	I	I	T
	138	Q	Q	Q	Q	L	Q	Q	Q	Q
	140	A	A	A	A	T	T	T	T	A
	141	P	P	P	P	P	P	P	P	S
	181	S	S	S	S	S	S	S	S	P
	195	T	T	T	T	T	T	T	T	D
	198	I	I	I	I	I	I	I	I	V
	236	D	D	D	D	N	N	N	N	N
	268	G	G	G	G	G	G	G	G	E
	282	V	V	V	V	V	V	V	V	I
	374	I	I	I	I	I	I	I	I	V
	490	Q	Q	Q	Q	Q	Q	Q	Q	K
	522	A	A	A	A	V	V	V	V	V
	531	M	M	M	M	M	M	M	M	I
	532	M	M	M	M	M	V	V	V	V
	549	I	I	I	I	V	I	I	I	I
	CS	REKRRKR↓GLF	REKRRKR↓GLF	REKRRKR↓GLF	REKRRKR↓GLF	REKRRKR↓GLF	REKRRKR↓GLF	REKRRKR↓GLF	REKRRKR↓GLF	RERRRKR↓GLF
	RBS	QRG	QRG	QRG	QRG	QRG	QRG	QRG	QRG	QRG
**NP**	16	S	S	S	G	G	G	G	G	G
	50	S	S	S	S	S	S	S	N	S
	51	D	D	D	D	D	D	D	D	N
	105	V	V	V	V	V	V	V	I	M
	129	S	S	S	A	A	A	A	T	A
	201	V	V	V	V	I	I	I	I	I
	219	F	F	F	F	Y	Y	Y	Y	Y
	371	I	I	I	I	M	M	M	M	M
	374	M	M	I	M	M	M	M	M	M
	452	R	R	R	K	R	R	R	K	K
	460	G	G	G	G	S	G	G	G	G
	492	N	N	N	N	N	N	N	N	S
**NA**	Stalk del.	No	No	No	No	No	No	No	No	No
	9	A	A	A	A	A	A	A	A	V

	28	L	L	L	L	V	V	V	V	V
	29	N	S	S	S	S	S	S	S	S
	33	M	M	M	M	M	M	M	M	T
	37	L	L	L	L	L	P	L	L	L
	47	K	K	K	K	K	K	K	K	N
	50	I	I	I	I	I	I	I	I	V
	51	I	I	I	V	V	V	V	V	V
	70	S	S	S	S	S	S	N	N	S
	71	V	V	V	V	A	V	V	V	V
	73	E	E	E	E	K	E	E	E	E
	82	A	A	A	A	A	A	A	A	T
	107	I	I	I	I	V	V	V	V	V
	114	F	F	F	F	F	Y	F	F	F
	137	A	A	A	A	A	A	A	A	S
	181	T	T	T	T	A	T	T	T	T
	202	I	I	I	I	V	V	V	V	V
	205	V	V	V	V	I	V	V	V	V
	214	I	I	I	I	V	V	V	V	V
	246	S	S	S	S	A	A	A	A	A
	264	D	D	D	D	D	D	D	D	G
	265	Q	Q	Q	Q	Q	Q	Q	Q	R
	266	A	A	A	A	T	T	T	T	T
	274	H	H	H	H	H	H	H	H	H
	292	R	R	R	R	R	R	R	R	R
	296	I	I	I	M	T	T	T	T	T
	304	V	V	V	V	V	V	V	V	I
	330	A	A	A	A	A	A	A	A	T
	360	M	M	M	M	V	V	V	V	V
	398	L	L	L	L	L	L	L	L	S
	451	H	H	H	H	H	H	H	H	Y
	470	G	G	G	G	K	K	K	K	K
	471	M	M	M	M	M	M	M	M	T
**M1**	15	V	V	V	V	V	V	V	V	I

	30	D	D	D	D	D	D	D	D	D
	33	A	A	A	A	V	V	V	V	A
	41	A	A	A	A	A	A	A	T	A
	42	L	L	L	L	I	L	L	L	L
	83	A	A	A	A	S	A	A	A	A
	95	R	R	R	R	R	R	R	R	K
	101	R	R	R	R	R	R	R	R	K
	120	T	T	T	T	T	T	T	A	T
	133	K	K	K	K	K	K	K	R	R
	139	A	A	A	A	T	T	T	T	T
	143	L	L	L	L	L	L	L	L	F
	165	I	I	I	I	I	I	I	I	M
	168	T	T	T	T	T	I	I	T	I
	200	A	A	A	A	A	A	A	A	S
	205	V	V	V	V	I	V	V	V	V
	207	S	S	S	S	S	S	S	S	N
	214	Q	Q	Q	Q	H	Q	Q	Q	Q
	218	T	T	T	T	T	T	T	T	T
	224	S	S	S	S	S	S	S	S	N
	232	D	D	D	D	D	D	D	D	N
	242	K	K	K	K	K	K	K	K	N
	248	L	L	L	L	M	M	M	M	M
**M2**	12	K	K	K	K	R	R	R	R	R
	13	N	N	N	N	N	N	N	N	T
	14	G	G	G	G	G	E	G	G	E
	18	N	N	N	N	K	K	K	K	R
	23	S	S	S	S	N	S	S	S	S
	28	I	I	I	I	I	I	I	I	V
	31	S	S	S	S	S	S	S	S	N
	35	I	I	I	I	V	I	I	I	I
	51	V	V	V	V	V	V	V	V	I
	54	R	R	R	R	R	C	R	R	R
	61	R	R	R	R	R	R	R	R	I

	65	T	T	T	T	T	A	T	T	T
**NS1**	del.	218-237 aa	218-237 aa	218-237 aa	218-237 aa	218-237 aa	218-237 aa	218-237 aa	218-237 aa	No
	3	P	P	P	P	S	S	S	S	S
	6	M	M	M	M	M	M	M	M	V
	7	L	L	L	L	L	L	L	L	S
	17	Y	Y	Y	Y	Y	Y	Y	Y	H
	20	K	K	K	K	R	K	K	K	K
	42	S	S	S	S	S	S	S	S	S
	47	G	G	G	G	G	S	G	G	G
	48	T	T	T	T	N	N	N	N	S
	53	S	S	S	S	G	G	G	G	D
	56	A	A	A	A	T	T	T	T	T
	60	E	E	E	E	A	A	A	A	A
	64	I	I	I	I	I	I	I	I	T
	65	V	V	V	V	V	V	V	V	L
	66	K	K	K	K	E	E	E	E	E
	70	K	K	K	K	E	E	E	E	G
	80	T	T	T	T	T	T	T	T	N
	81	V	V	V	V	V	V	V	V	I
	84	G	G	G	G	S	S	S	S	V
	86	S	S	S	S	S	S	S	S	T
	92	D	D	D	D	D	D	D	D	D
	103	F	F	F	F	F	F	F	F	F
	106	M	M	M	M	M	M	M	M	M
	109	R	R	R	R	Q	Q	Q	Q	Q
	114	P	P	P	P	S	S	S	S	S
	127	N	N	N	N	N	N	N	N	T
	139	N	N	N	N	N	N	N	N	D
	143	A	A	A	A	A	A	A	A	T
	166	F	F	F	F	F	F	F	F	L
	176	N	N	N	N	N	N	N	N	I
	189	N	N	N	N	D	D	D	D	D
	202	A	A	A	A	A	A	A	A	T

	205	N	N	N	N	N	N	N	N	S
	207	D	D	D	D	N	N	N	N	N
	216	P	P	P	P	P	P	P	P	S
**NS2**	3	P	P	P	P	S	S	S	S	S
	6	M	M	M	M	M	M	M	M	V
	7	L	L	L	L	L	L	L	L	S
	19	M	M	M	M	M	M	M	M	L
	27	G	G	G	G	D	D	D	D	G
	31	I	I	I	I	M	M	M	M	M
	36	G	G	G	G	G	G	G	G	E
	48	T	T	T	T	T	T	T	T	A
	60	S	S	S	S	S	S	S	S	N
	70	G	G	G	G	G	G	G	G	S
	83	V	V	V	M	V	V	V	V	M

Abbreviations: aa: amino acids; Ak: Astrakhan; Ck: chicken; CS: cleavage site; del.: deletion; NL: Netherlands; RBS: receptor-binding site (residues 222~224 in the H5 numbering or 226~228 in the H3 numbering); RoD: Rostov-on-Don; V: Voronezh.

Any residue differences in the nine viruses are presented. The residues are marked in red, if they are mutated (1) only in the human virus or (2) in three Astrakhan viruses in 2020 or (3) in all four viruses in 2020.
